# 

**Published:** 1858-01

**Authors:** 


### CONTENTS.

Original Communications— Page.

An Address read before the Esculapian Society of the Wabash Valley. By E. Read, M.D. .......... 5

Cases treated at the College Clinic by Prof. Brainard. Reported by Edwin Powell, M.D. .......... 28

Report of Cases in Obstetrical Practice. By Thomas Bevan, M.D. .......... 32

Translations for the Journal. By Dr. Byford.

Leucorrhea of Children .......... 36

Resume of the Results of Dr. Friedrich's Researches on Abdominal Typhus (Typhoid) Fever in Children .......... 38

Mixture for the Teeth .......... 41

Book Notices .......... 41 to 48

Editorial—

Important Correction .......... 48

Prolific .......... 49

Glycerine in Phthisis .......... 49

To Correspondents .......... 50

Homœopathy and the Chicago City Hospital .......... 50

Hymenial .......... 50

Advertisements .......... 50, 51, 52

